# Bacteriophage ISP eliminates *Staphylococcus aureus* in planktonic phase, but not in the various stages of the biofilm cycle

**DOI:** 10.1038/s41598-024-65143-9

**Published:** 2024-06-22

**Authors:** Mariëlle Verheul, Aat A. Mulder, Sven C. J. van Dun, Maia Merabishvili, Rob G. H. H. Nelissen, Mark G. J. de Boer, Bart G. Pijls, Peter H. Nibbering

**Affiliations:** 1 Department of Infectious Diseases, Leiden University Medical Center, 2300RC Leiden, The Netherlands; 2 Department of Orthopedics, Leiden University Medical Center, 2300RC Leiden, The Netherlands; 3 Department of Cell and Chemical Biology, Electron Microscopy Facility, Leiden University Medical Center, 2300RC Leiden, The Netherlands; 4 Laboratory for Molecular and Cellular Technology, Queen Astrid Military Hospital, Brussels, Belgium

**Keywords:** Biofilms, Bacteriophages, Bacterial infection

## Abstract

Metal-implant associated bacterial infections are a major clinical problem due to antibiotic treatment failure. As an alternative, we determined the effects of bacteriophage ISP on clinical isolates of *Staphylococcus aureus* in various stages of its life cycle in relation to biofilm formation and maturation. ISP effectively eliminated all planktonic phase bacteria, whereas its efficacy was reduced against bacteria attached to the metal implant and bacteria embedded within biofilms. The biofilm architecture hampered the bactericidal effects of ISP, as mechanical disruption of biofilms improved the efficacy of ISP against the bacteria. Phages penetrated the biofilm and interacted with the bacteria throughout the biofilm. However, most of the biofilm-embedded bacteria were phage-tolerant. In agreement, bacteria dispersed from mature biofilms of all clinical isolates, except for LUH15394, tolerated the lytic activity of ISP. Lastly, persisters within mature biofilms tolerated ISP and proliferated in its presence. Based on these findings, we conclude that ISP eliminates planktonic phase *Staphylococcus aureus* while its efficacy is limited against bacteria attached to the metal implant, embedded within (persister-enriched) biofilms, and dispersed from biofilms.

## Introduction

Prosthetic joint infections (PJI) are a major challenge to patients, clinicians and orthopedic surgeons as current treatment, i.e., surgical debridement followed by antibiotics, fails in 30–40% of cases^1^. To eliminate the infection, other strategies like two-stage revision surgery are required, which massively impacts the patients’ mobility, creates psychological distress, results in high healthcare costs and is associated with a higher risk of mortality^2–4^. Moreover, failure of treatment with antibiotics can result in development of antibiotic resistance in the remaining bacterial population, which further contributes to the worldwide pandemic of antimicrobial resistance (AMR)^5,6^.

In addition to AMR, biofilm- and persister formation are responsible for the reduced efficacy of antibiotics. *Staphylococcus aureus* (*S*. *aureus*) is most frequently associated with PJI and the beforementioned treatment failure^1,7^. Briefly, bacteria adhere to the implant material and form microcolonies that start producing extracellular matrix (ECM) composed of exopolysaccharides, extracellular DNA (eDNA) and proteins. The biofilm matures over time and in this dynamic process, single bacteria within the biofilm may disperse into the surrounding tissue^8^. The dense biofilm matrix often hampers the penetration of environmental stressors like host immune cells and antibiotics^9^. Other mechanisms that contribute to antibiotic resistance and tolerance of bacteria within biofilms include the expression of efflux pumps by bacteria, horizontal gene transfer and quorum sensing^10^. Gradients of nutrients, oxygen and pH within the biofilm result in heterogenous populations with varying metabolic activities and thus different susceptibilities to antimicrobials^11^. For example, bacteria can undergo a phenotypic switch to metabolic dormancy under external stressors like antibiotics, resulting in their survival^12^. Besides their low metabolic activity, these persisters have shown to express high levels of efflux pumps, which further mediates their tolerance against antibiotics^13^. After cessation of antibiotic treatment, persisters become metabolically active again and induce infection relapse^14^.

This not only underscores the limitation of extensive antibiotic use and surgical treatment, but also stresses research into different modalities for treatment of PJI. For that matter, lytic bacteriophages (phages) are an interesting approach as they infect bacterial species with high efficacy and specificity, while leaving the patient’s microbiome intact^15,16^. Therefore, phages have to be adapted to the bacterial host for clinical use. However, the host range of phages against bacterial strains within a species can be broad. For example phage ISP, which is a component of clinical phage cocktail BFC-1^17^, induced bacterial lysis in 86% of the tested *S. aureus* clinical strains in vitro, which is promising for its clinical application against many *S. aureus*-induced infections^18^. Phages can be preadapted to their host by co-evolving, which improves their efficacy and limits the development of resistance by bacteria^19,20^. Another advantage of phages is that development of personalized phage treatment against bacterial infections requires only days to weeks compared to years or decades for the development of new antibiotics^21^.

In the last decades, many clinical cases of bone and joint infections have indicated the ability of phages to improve the efficacy of antibiotic treatment against antibiotic-resistant bacteria, without serious adverse events related to the administration of phages^22–24^. Nevertheless, the absence of suitable controls hampers the interpretation on the efficacy of phages themselves. Most in vitro studies have focused on the effects of phages against bacteria within 24–48 h biofilms, whilst in the clinic the biofilm is most probably at least 7 days old. In general, it takes several days before patients have treatment after presenting with symptoms of an infected implant, which means that the biofilm has matured and is no longer in its first developing stage. Previous research has also indicated that at least 5 days of biofilm culturing is required to obtain the maximum quantity of *S. aureus* biofilms on titanium surfaces in vitro ^25^. In addition, 7 days culturing of bacterial biofilms resulted in low pH and hypoxic regions, which are associated with mature biofilms and its heterogenous bacterial subpopulations^26^.

Knowledge is limited on the effect of phages as single agent against bacteria in various stages related to biofilm formation- and maturation on metal implants. Hence, this in vitro study aimed to determine the effects of phage ISP against *S. aureus* in different stages of its life cycle, including: planktonic phase (stage I), after bacterial attachment (stage II), within immature biofilms (stage III), within and dispersed from mature biofilms (stage IV-a, stage IV-b) and within persister-enriched mature biofilms (stage V).

## Material and methods

### Bacteria and bacterial culture

The clinical isolates of *S. aureus* in the present study were methicillin-resistant *S. aureus* (MRSA) (LUH14616, sequence type 247, NCCB 100829; Department of Medical Microbiology, Maastricht University Medical Center, Maastricht, The Netherlands) and three methicillin-sensitive *S. aureus* (MSSA) isolated from patients with prosthetic joint infection (PJI), coded as LUH15393, LUH15394 and LUH15395 (Department of Medical Microbiology, Leiden University Medical Center, Leiden, The Netherlands). Bacterial strains were frozen in 20% glycerol and stored at − 80 °C. Prior to experiments, these stocks were spread on trypticase soy agar plates with 5% sheep blood and incubated overnight at 37 °C. Colonies from this plate were cultured in tryptone soy broth (TSB) to reach a mid-logarithmic phase in 2.5 h or stationary phase in 24 h at 200 rpm and 37 °C. Next, bacteria were obtained by centrifugation (1000×*g* for 10 min) and washed with phosphate-buffered saline (PBS, pH 7.4). Based on the optical density at 600 nm, bacteria were diluted to the desired concentration in medium.

### Antibiotics

Stock solutions of rifampicin (4 g/L in DMSO) and ciprofloxacin (25.6 g/L in milli-Q water) were stored at − 20 °C until use. Next, rifampicin and ciprofloxacin were pre-diluted in milli-Q (100× and 20×, respectively) and then further diluted to 10 mg/L and 1280 mg/L, respectively, in brain heart infusion (BHI) broth.

### Phage propagation and enumeration

Fresh stocks of ISP were propagated using the double agar overlay method^27^, by mixing 100 µL of ISP at 10^6^ PFU/mL and 100 µL of mid-logarithmic MRSA at 10^9^ CFU/mL in 3.5 mL of semi-solid Luria Bertani (LB) agar at 45 °C to pour on LB agar plates for incubation overnight at 37 °C. The semi-solid layer was transferred into a 50 mL tube, vortexed and centrifuged for 20 min at 4 °C at 6000×*g*. The supernatant was filtered with a 0.45 µm filter. Phage titers were determined in triplicate by counting the plaque forming units (PFU)/mL after overnight incubation of MRSA with the phage dilutions using the double agar overlay method^27^. The lytic activity of ISP against the various bacterial strains was assessed by a spot test and the double agar overlay method^27^. Briefly, 100 µL of mid-logarithmic MRSA at 10^9^ CFU/mL were mixed with 3.5 mL of semi-solid LB agar 45 °C and poured on LB plates. Next, 10 µL of 100-fold dilutions of ISP at 10^10^ PFU/mL were spotted on the plate. Plates were inverted and incubated overnight at 37 °C. The appearance of clear plaques indicated phage-induced bacterial lysis and thus bacterial sensitivity against the phage. The efficiency of plating (EOP), i.e., the phage titer on a test strain compared to the titer of the reference strain (LUH14616)^28^, was determined by four independent experiments and amounted to 0.81 (LUH15393), 1.11 (LUH15394), and 0.58 (LUH15395). Finally, phage stocks were stored at 4 °C until use.

### Exposure of experimental models to phages

The effects of phage ISP were investigated against experimental models representing the different stages of the *S. aureus* life cycle, as summarized in Fig. 6 (Created using BioRender.com).

#### Phage exposure of planktonic bacteria (stage I)

Mid-logarithmic phase MRSA and MSSA at a final concentration of 10^6^ CFU/mL were mixed with ISP at final concentrations ranging from 10^1^–10^8^ PFU/mL in BHI broth in a 96-wells v-bottom polypropylene plate, while bacteria mixed with BHI served as controls. Stationary phase MRSA at a final concentration of 10^6^ CFU/mL were exposed to ISP at final concentrations ranging from 10^5^–10^8^ PFU/mL in 0.9% saline supplemented with 2% TSB. Plates were sealed with non-breathable plastic sealing film and incubated at 37 °C at 85 rpm for 24 h. Of note, phage activity was not affected by sealing plates with the non-breathable plastic sealing film when compared to the breathable rayon sealing film (Supplementary Fig. S1). Afterwards, the bacterial suspensions were mixed with 10% ammonium iron (II) sulfate hexahydrate (FAS, 100 mM, v/v) to neutralize residual activity of extracellular phages^29^, and plated on Mueller Hinton (MH) agar plates to assess the bacterial counts microbiologically. Any possible contamination was detected by the use of medium controls. Results are expressed as log CFU/mL.

#### Phage exposure of bacteria after attachment to the implant mimic (stage II)

Second, 100 µL of mid-logarithmic phase MRSA and MSSA at 10^7^ CFU/mL in BHI broth was added on top of medical grade titanium-7% aluminum-6% niobium [TAN; iso5832/11 discs, handled as described previously^30^] in 96-wells flat-bottom polystyrene plates. After 1 h, TAN discs were washed twice with 0.9% saline to remove non-adherent bacteria and transferred to a 96-wells flat-bottom polystyrene plate with 100 µL of ISP at final concentrations ranging from 10^1^–10^8^ PFU/mL. BHI served as control. The plate was sealed with non-breathable plastic sealing film and incubated for 24 h at 37 °C in a humidified environment. Non-biofilm associated bacteria were removed by two washes with 0.9% saline and TAN discs were transferred to a 96-wells flat-bottom polystyrene plate for sonication [10 min at 40 kHz; Bransonic^®^ M mechanical bath 1800 (Branson Ultrasonics, Brookfield, CT, USA)] in 0.9% saline with 10 mM FAS to assess the bacterial counts microbiologically.

#### Phage exposure of bacteria within biofilms (stage III, IV-a and IV-b)

MRSA and MSSA in mid-logarithmic phase at a concentration of 10^7^ CFU/mL were added on top of TAN discs as described above. Immature and mature biofilms were obtained by sealing these plates with non-breathable plastic sealing film or breathable rayon sealing film and incubation for 24 h or 7 days at 37 °C in a humidified environment, respectively. Next, non-biofilm associated bacteria were removed from biofilms by two washes and TAN discs were transferred to a fresh plate with ISP (ranging from 10^5^–10^8^ PFU/mL) in BHI, while BHI served as control. Plates were sealed, incubated, washed after incubation and transferred to a fresh plate for sonication, for enumeration of bacterial counts as mentioned before. To determine the effects of ISP against bacteria from mechanically disrupted immature and mature biofilms, such biofilms were sonicated in BHI prior to the addition of ISP in 96-wells v-bottom polypropylene plates and 24 h incubation (85 rpm, 37 °C). To determine the effect of ISP against bacteria dispersed from mature biofilms, non-biofilm associated bacteria were washed from 10 biofilms and discs with biofilms were transferred to a new plate with fresh BHI. After 24 h, the supernatant of the biofilms containing dispersed bacteria was pooled, brought to a final concentration of 10^6^ CFU/mL and incubated with ISP at a final concentration of 10^8^ PFU/mL in BHI as described for planktonic bacteria.

#### Phage exposure of persister-enriched mature MRSA biofilms (stage V)

Mature MRSA biofilms on TAN discs were enriched for persisters by exposure to high doses of antibiotics (10 mg/L rifampicin and 1280 mg/L ciprofloxacin) for at least three consecutive days, as described before^31^. Biofilms were washed prior to and after each exposure and controls received the diluent of antimicrobials in BHI. After persister-enrichment of mature MRSA biofilms, TAN discs were transferred to a fresh plate with ISP (concentrations ranging from 10^5^–10^8^ PFU/mL in BHI). As described earlier, persister-enriched mature biofilms were sonicated for microbiological determination of the viable bacterial counts. To determine the effects of ISP against persisters from mechanically disrupted persister-enriched mature MRSA biofilms, such biofilms were sonicated in BHI before mixing with ISP at final concentrations ranging from 10^5^–10^8^ PFU/mL in BHI and 24 h incubation.

### Transmission electron microscopy (TEM) of biofilms

For TEM analysis of biofilms, mature MRSA biofilms were cultured as described above on 0.4 µm 12-well transparent ThinCert^™^ membranes (Greiner Bio-One, #21210116). Biofilms were exposed to ISP at a concentration of 10^8^ PFU/mL in BHI for 24 h while controls received BHI. Samples were fixed in 3% glutaraldehyde in 0.2 M cacodylate buffer (double concentrated 1:1) for 1 h at RT, rinsed carefully with 0.1 M cacodylate, postfixed in 1% osmium tetroxide/1.5% potassium hexacyanoferrate (III)/0.1 M cacodylate buffer for 1 h on ice, rinsed carefully with 0.1 M cacodylate and subsequently incubated overnight in 70% ethanol. Next, samples were cut out of the insert into small pieces for infiltration with EPON (Lx112). Briefly, samples were dehydrated in increasing concentrations of ethanol up to 100%, thereafter in increasing concentrations of EPON in ethanol and in pure ethanol. Sample pieces were put in a mold for polymerization at 60 °C for 48 h, then 90 nm sections were made with an ultramicrotome and put on one-hole grids with a layer of carbon coated pioloform. Sections were contrasted with 7% uranyl acetate and lead hydroxide according to Reynolds. Sections were examined with a Tecnai 12Twin equipped with a Gatan one-view camera on binning 2. Overlapping images were stitched together as described previously^32^, and analyzed using Aperio ImageScope 12.4.6.

### Data analysis

Data on log CFU/mL is highly skewed and log-transformation was required to approximate a normal distribution. This allowed calculation of the mean log CFU/mL with 95% confidence intervals as previously recommended^33,34^. GraphPad Prism 9.3.1 was used for log-transformation of the data and calculating the 95% confidence intervals.

## Results

### Effect of ISP on planktonic phase *S. aureus*

**Figure 1:**
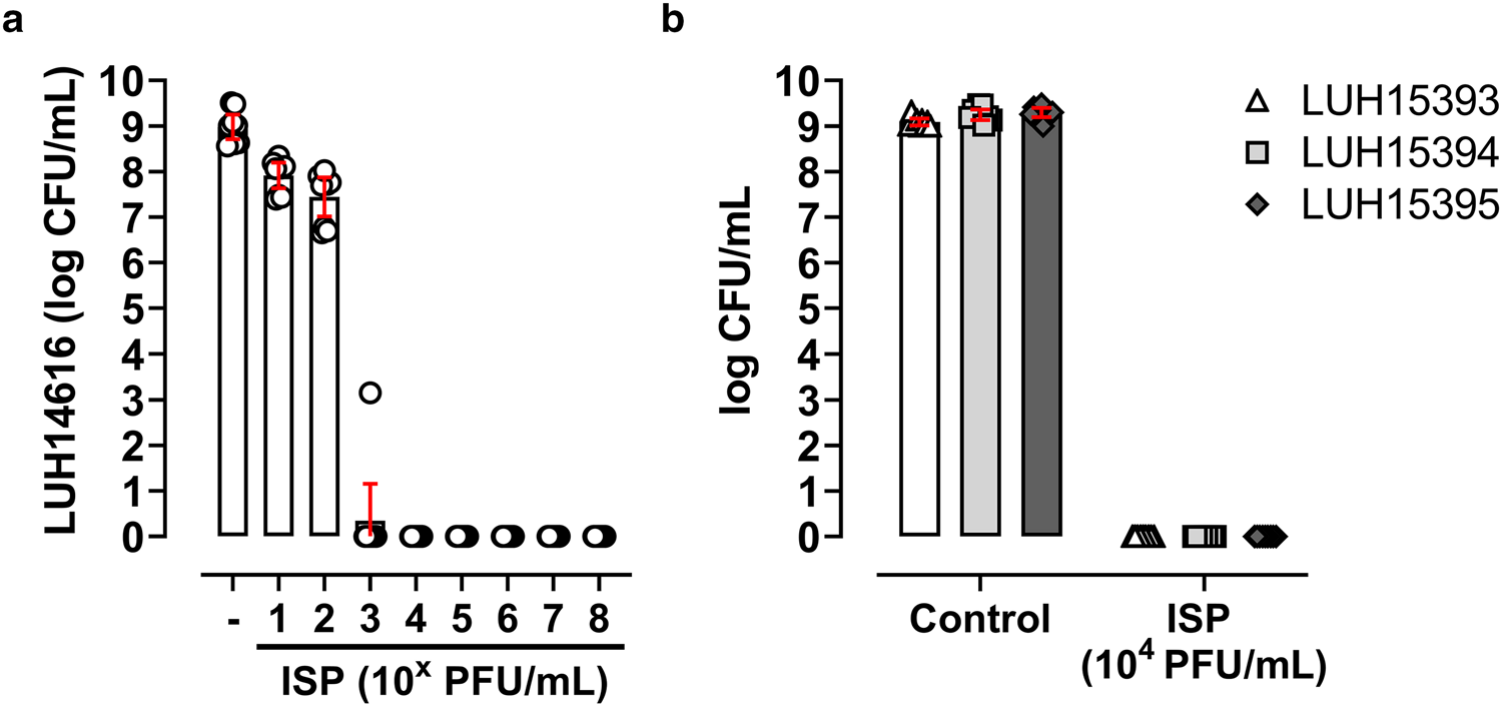
Effect of ISP against planktonic *S*. *aureus*. Planktonic (stage I) (**a**) methicillin resistant *S*. *aureus* (MRSA) or (**b**) methicillin-sensitive *S*. *aureus* (MSSA; LUH15393, LUH15394, LUH15395) in mid-logarithmic phase at a final concentration of 10^6^ CFU/mL were exposed to phage ISP at final concentrations (**a**) ranging from 10^1^–10^8^ plaque forming units (PFU)/mL (multiplicity of infection (MOI) 10^−5^–100) or (**b**) 10^4^ PFU/mL (MOI 0.01) in brain–heart infusion (BHI) broth. Residual phage activity was neutralized by the addition of 10 mM ammonium iron (II) sulfate hexahydrate (FAS) after 24 h phage exposure and before microbiological determination of viable bacteria in CFU/mL. Results are from three independent experiments each in triplicate. The mean CFU/mL and the 95% confidence intervals of the log-transformed data are indicated by the bar and the error bars, respectively.

First, we explored the effect of ISP at final concentrations ranging from 10^1^–10^8^ plaque forming units (PFU)/mL (multiplicity of infection (MOI) 10^−5^–100) on planktonic methicillin-resistant *S. aureus* (MRSA; LUH14616) in mid-logarithmic phase at a final concentration of 10^6^ CFU/mL. Results revealed that MRSA were dose-dependently eliminated by ISP at final concentrations ≥ 10^4^ PFU/mL (MOI ≥ 0.01) completely eradicating the bacteria after 24 h (Fig. 1a). In addition, stationary phase MRSA at a final concentration of 10^6^ CFU/mL were reduced with a mean 3.9-log CFU/mL by ISP at a final concentration of 10^6^ PFU/mL (MOI 1), and totally eliminated by ISP with final concentrations ≥ 10^7^ PFU/mL (MOI ≥ 10) (Supplementary Fig. S2). Next, we assessed the efficacy of ISP on clinical isolates derived from patients with prosthetic joint infection (PJI), using the lowest phage concentration eradicating mid-logarithmic phase MRSA (i.e., 10^4^ PFU/ml). In line with our observations for the MRSA strain, all three methicillin-sensitive *S*. *aureus* (MSSA; LUH15393-LUH15395) strains at a final concentration of 10^6^ CFU/mL were eliminated by ISP at a final concentration of 10^4^ PFU/mL (MOI 0.01) (Fig. 1b). Altogether, ISP was highly effective against all tested *S*. *aureus* strains in planktonic phase.

### Effect of ISP on *S. aureus* attached to the implant surface

**Figure 2:**
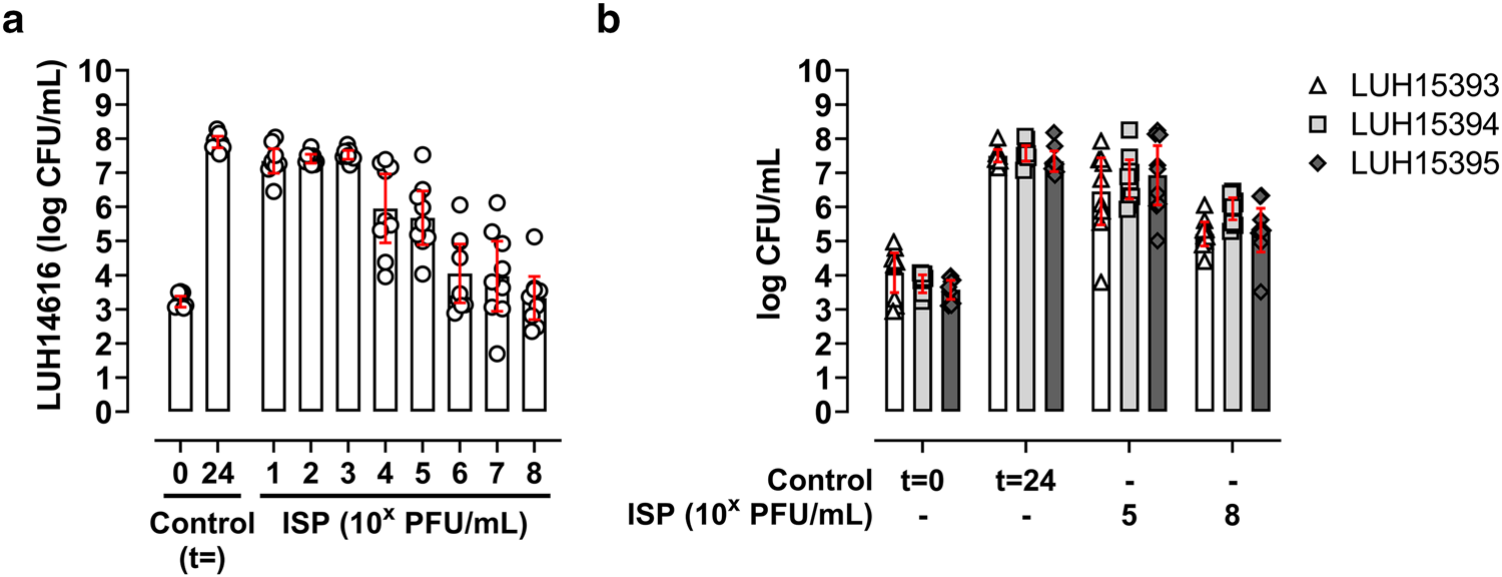
Effect of ISP on *S*. *aureus* attached to the implant mimic. (**a**) Methicillin-resistant *S*. *aureus* (MRSA; LUH14616) or (**b**) methicillin-sensitive *S. aureus* (MSSA; LUH15393, LUH15394, LUH15395) in mid-logarithmic phase attached to titanium-aluminum-niobium (TAN) discs in 1 h (stage II) were subsequently exposed to ISP at concentrations (**a**) ranging from 10^1^–10^8^ plaque forming units (PFU)/mL (multiplicity of infection (MOI) 0.01—10^5^) or (**b**) 10^5^ and 10^8^ PFU/mL (MOI 100 and 10^5^) in brain–heart infusion (BHI) broth for 24 h. Residual phage activity was neutralized by the addition of 10 mM ammonium iron (II) sulfate hexahydrate (FAS) before microbiological determination of viable bacteria by CFU/mL. Results are from three independent experiments each in triplicate. Bacteria attached to TAN discs after 1 h incubation are shown at t = 0, bacteria incubated for 24 h in BHI are shown at t = 24. The mean CFU/mL and the 95% confidence intervals of the log-transformed data are indicated by the bar and the error bars, respectively.

Second, we investigated the ability of ISP to kill bacteria attached to metal implant mimics (i.e., titanium–aluminum–niobium (TAN) discs) aimed at preventing bacterial growth towards a biofilm. After 1 h incubation, a mean 3.2-log CFU/mL (95% CI 3.1–3.4) MRSA (LUH14616) were attached to TAN discs. Exposure of these bacteria to ISP at 10^5^ PFU/mL (MOI 100) for 24 h resulted in an increased bacterial load, whereas this bacterial outgrowth was prevented by ISP at 10^8^ PFU/mL (MOI 10^5^) (Fig. 2a, Supplementary Table S1). Similarly, the efficacy of ISP at 10^5^ (MOI 100) and 10^8^ PFU/mL (MOI 10^5^) was assessed against LUH15393, LUH15394 and LUH15395 attached to the metal implant mimic, corresponding to a mean 4.1-log (95% CI 3.5–4.7), 3.7-log (95% CI 3.5–4.0), 3.6-log (95% CI 3.3–3.8) CFU/mL, respectively (Fig. 2b, Supplementary Table S1). Results showed a mean increase of 1.1-log (LUH15393), 2.2-log (LUH15394) and 1.7-log (LUH15395) CFU/mL in the presence of ISP at 10^8^ PFU/mL (MOI 10^5^) (Fig. 2b, Supplementary Table S1). Of note, the bacterial counts were still 2.3-log (LUH15393), 1.6-log (LUH15394) and 2-log (LUH15395) CFU/mL lower compared to the control without ISP. Also, ISP at 10^5^ PFU/mL (MOI 100) was less effective than 10^8^ PFU/mL (MOI 10^5^) in preventing outgrowth of MSSA after bacterial attachment (Fig. 2b). Thus, ISP could not eliminate MRSA and MSSA attached to the implant mimic, while hampering the bacterial outgrowth.

### Effect of ISP on *S. aureus* in immature biofilms

**Figure 3:**
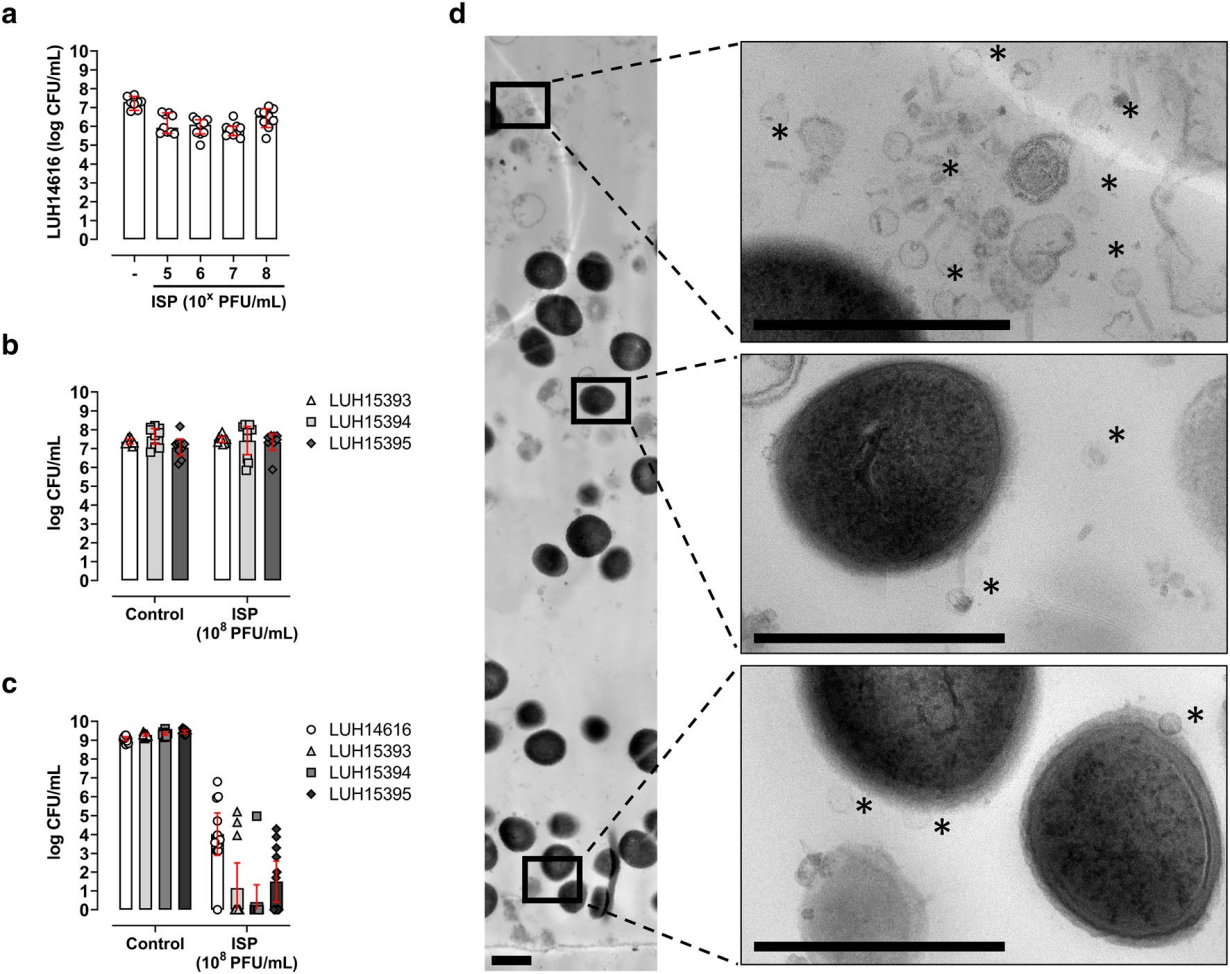
Effect of ISP on *S*. *aureus* within and from immature biofilms. Immature biofilms (stage III) of (**a**) methicillin-resistant *S*. *aureus* (MRSA; LUH14616) and (**b**) methicillin-sensitive *S*. *aureus* (MSSA; LUH15393, LUH15394 and LUH15395) on titanium–aluminum–niobium (TAN) discs were exposed to ISP at concentrations (**a**) ranging from 10^5^–10^8^ plaque forming units (PFU)/mL (multiplicity of infection (MOI) 0.01–10) or (**b**) 10^8^ PFU/mL (MOI 10) in brain–heart infusion (BHI) broth for 24 h. Biofilms on TAN discs were sonicated in 0.9% saline and 10 mM ammonium iron (II) sulfate hexahydrate (FAS) to neutralize residual phage activity and determine the bacterial load microbiologically in CFU/mL. Immature MRSA and MSSA biofilms in (**c**) were mechanically disrupted prior to 24 h phage exposure at a final concentration of 10^8^ PFU/mL (MOI 10) and the residual phage activity was neutralized by supplementing with 10 mM FAS before microbiological determination of the bacterial load. Results are from (**a**,**b**) three and (**c**) four independent experiments each in triplicate. The mean CFU/mL and the 95% confidence intervals of the log-transformed data are indicated by the bar and the error bars, respectively. Cross-sectional transmission-electron microscopy of immature MRSA biofilms exposed to ISP at 10^8^ PFU/mL (MOI 10) for 24 h is visualized in (**d**). The apical layer of the biofilm is depicted on top, the basal layer of the biofilm on the insert on the bottom. (Regions with) phages are indicated by *, the bar indicates 1 µm.

Third, the effect of ISP (10^5^–10^8^ PFU/mL; MOI 0.01–10) against bacteria residing within immature biofilms on TAN discs was determined. Results did not show a dose-dependent elimination, but a minor reduction of 0.9-log CFU/mL MRSA (LUH14616) within immature biofilms upon exposure to ISP at 10^8^ PFU/mL (MOI 10) (Fig. 3a, Supplementary Table S2). Based on this finding, ISP at 10^8^ PFU/mL (MOI 10) was tested against MSSA within immature biofilms. Bacterial counts of LUH15393, LUH15394, LUH15395 within immature biofilms were not affected by ISP (Fig. 3b, Supplementary Table S2). To investigate the direct effect of ISP on bacteria originating from the biofilm, we determined the effect of ISP on MRSA and MSSA obtained by mechanical disruption of immature biofilms. Interestingly, the efficacy of ISP at a final concentration of 10^8^ PFU/mL (MOI 10) drastically improved on bacteria from disrupted biofilms, resulting in a mean reduction of 3.3-log MRSA (LUH14616), 6.2-log (LUH15393), 7.2-log (LUH15394) and 5.6-log (LUH15395) CFU/mL compared to the biofilm control (Fig. 3c, Supplementary Table S2). To investigate whether ISP was able to penetrate the biofilm matrix, immature MRSA (LUH14616) biofilms were exposed to ISP at 10^8^ PFU/mL (MOI 10) and thereafter visualized cross-sectionally with TEM. Results showed the presence of phages and their interaction with bacteria in the various layers of the biofilm (Fig. 3d). Thus, although ISP could penetrate the different biofilm layers, the efficacy of ISP against bacteria within immature biofilms was limited due to the biofilm architecture.

### Effect of ISP on *S. aureus* in mature biofilms

**Figure 4:**
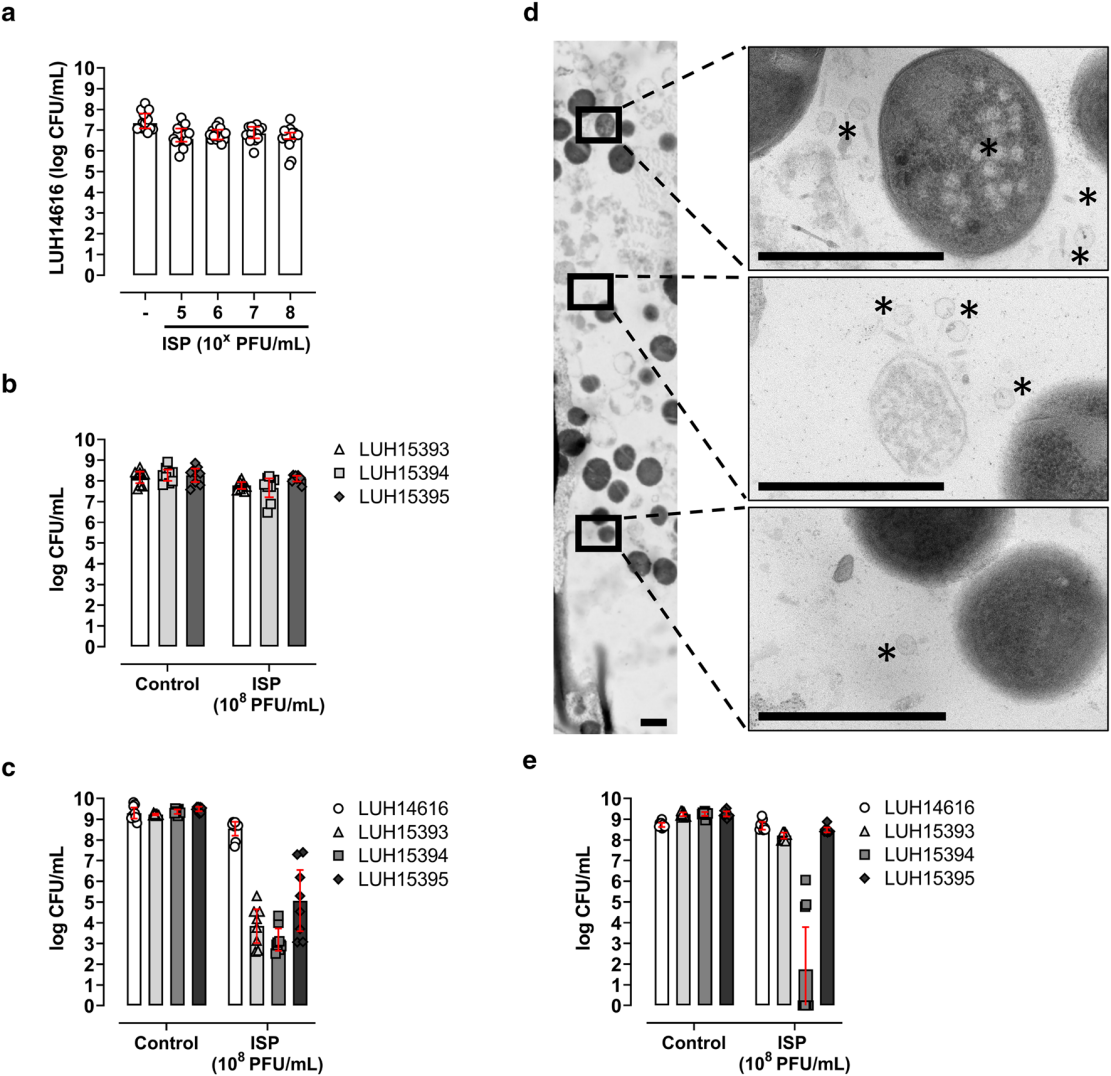
Effect of ISP on *S*. *aureus* within and from mature biofilms. Mature biofilms (stage IV-a) of (**a**) methicillin-resistant *S. aureus* (MRSA; LUH14616) and (**b**) methicillin-sensitive *S*. *aureus* (MSSA; LUH15393, LUH15394 and LUH15395) on titanium–aluminum–niobium (TAN) discs were exposed to phage ISP at concentrations (**a**) ranging from 10^5^–10^8^ plaque forming units (PFU)/mL (multiplicity of infection (MOI) 10^−3^–1) or (**b**) 10^8^ PFU/mL (MOI 1) in brain–heart infusion (BHI) broth for 24 h. Thereafter, biofilms on TAN discs were sonicated in 0.9% saline and 10 mM ammonium iron (II) sulfate hexahydrate (FAS) to neutralize residual phage activity and determine the bacterial load microbiologically (depicted as CFU/mL). Mature MRSA and MSSA biofilms in (**c**) were mechanically disrupted prior to 24 h ISP exposure at a final concentration of 10^8^ PFU/mL (MOI 1) followed by supplementing with 10 mM FAS and microbiological determination of the bacterial load. Cross-sectional transmission-electron microscopy of mature MRSA biofilms exposed to ISP at a concentration of 10^8^ PFU/mL (MOI 1) for 24 h is visualized in (**d**). The apical layer of the biofilm is depicted on top, the basal layer of the biofilm on the insert on the bottom. (Regions with) phages are indicated by *, bar indicates 1 µm. MRSA and MSSA (**e**) dispersed from mature biofilms (stage IV-b) were brought to a final concentration of 10^6^ CFU/mL and exposed to phage ISP at a final concentration of 10^8^ PFU/mL (MOI 100) in BHI for 24 h, followed by neutralizing the residual phage activity with 10 mM FAS and microbiological determination of the bacterial counts. Results are from (**a**) five and (**b**,**c**,**e**) three independent experiments each in (**a**,**b**,**e**) triplicate and (**c**) triplicate and duplicate. The mean CFU/mL and the 95% confidence intervals of the log-transformed data are indicated by the bar and the error bars, respectively.

As 7 days of biofilm culturing better represents clinically relevant mature *S. aureus* biofilms than 24 h biofilms in vitro^25,26^, we assessed the effect of ISP (10^5^–10^8^ PFU/mL; MOI 10^−3^–1) on MRSA (LUH14616) residing within mature, 7 day biofilms on TAN discs. As a result, ISP at 10^8^ PFU/mL (MOI 1) reduced MRSA within mature biofilms by 0.8-log CFU/mL (Fig. 4a, Supplementary Table S3). In addition, repeated exposure of mature MRSA biofilms to ISP at 10^8^ PFU/mL (MOI 1) for three consecutive days did not improve the phage’s efficacy (Supplementary Fig. S3). Yet, reversible phage tolerance was observed in the ISP-surviving MRSA population. That is, re-culturing the ISP-surviving population to mid-logarithmic phase resulted in phage-induced bacterial clearance, which could be prevented by using FAS to neutralize extracellular phages (Supplementary Fig. S4). Next, the effect of ISP at 10^8^ PFU/mL (MOI 1) was tested against the MSSA strains within mature biofilms. Results showed minor reductions of bacterial counts within mature biofilms upon exposure to ISP, i.e., 0.4-log, 0.6-log and 0.2-log CFU/mL of LUH15393, LUH15394 and LUH15395, respectively (Fig. 4b, Supplementary Table S3). To assess the direct effect of ISP on bacteria originating from mature biofilms, biofilms were mechanically disrupted and bacteria were thereafter exposed to ISP. MRSA (LUH14616) from disrupted biofilms were not affected by ISP at 10^8^ PFU/mL (MOI 1) (Fig. 4c, Supplementary Table S3). However, ISP at 10^8^ PFU/mL (MOI 1) reduced bacterial counts of LUH15393, LUH15394 and LUH15395 from mechanically disrupted biofilms by 4.3-log, 5.1-log and 3.2-log CFU/mL, respectively, compared to the biofilm control (Fig. 4c, Supplementary Table S3). Cross-sectional TEM analysis of mature MRSA biofilms revealed the presence of phages throughout the biofilm, also within biofilm-embedded bacteria (Fig. 4d). Further, phages retained their lytic activity within the biofilm (data not shown). In summary, the efficacy of ISP against mature biofilm-embedded MRSA and MSSA was restricted due to the intact biofilm architecture and a phage-tolerant phenotype.

### Effect of ISP on *S. aureus* dispersed from mature biofilms

Next, we investigated the ability of ISP to eliminate bacteria that dispersed in 24 h from mature biofilms. LUH15394 at a final concentration of 10^6^ CFU/mL was reduced with a mean 5.3-log CFU/mL by exposure to ISP at a final concentration of 10^8^ PFU/mL (MOI 100) for 24 h, while the other bacterial strains were not affected by ISP (Fig. 4e, Supplementary Table S4). To identify whether the observed recalcitrance of the other strains was reversible, we incubated the dispersed bacteria in brain–heart infusion (BHI) broth for 24 h before exposure to ISP. Indeed, results revealed that all MSSA strains at a final concentration of 10^6^ CFU/mL were eliminated by ISP at a final concentration of 10^8^ PFU/mL (MOI 100) (Supplementary Fig. S5). Of note, MRSA (LUH14616) required 48 h pre-incubation to reverse the phage-tolerance. Together, dispersed bacteria from mature LUH15394 biofilms were effectively reduced by ISP, while the dispersed bacteria from the other biofilm strains were phage-tolerant.

### Effect of ISP on persister-enriched mature *S. aureus* biofilms

**Figure 5:**
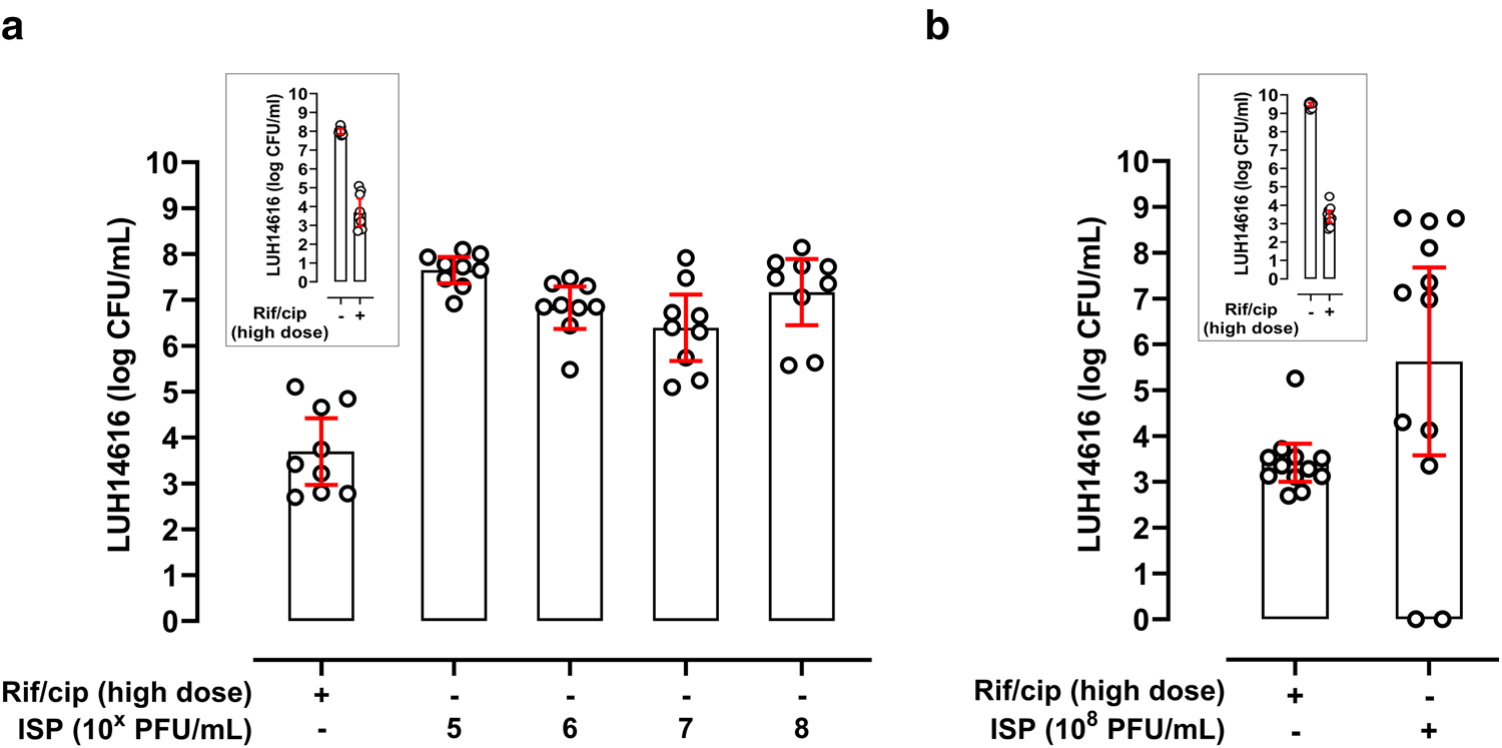
Effect of ISP on persisters within and originating from mature methicillin-resistant *S*. *aureus* biofilms. Methicillin-resistant *S. aureus* (MRSA; LUH14616) within mature biofilms on titanium-aluminum-niobium (TAN) discs were enriched for persisters (stage V) by exposure to high dose antibiotics (10 mg/L rifampicin and 1280 mg/L ciprofloxacin) for three consecutive days [inserts in (**a**,**b**)]. Thereafter, persister-enriched mature biofilms were exposed to ISP at final concentrations (**a**) ranging from 10^5^–10^8^ plaque forming units (PFU)/mL (multiplicity of infection (MOI) 100–10^5^) or (**b**) 10^8^ PFU/mL (MOI 10^5^) in brain–heart infusion broth (BHI) for 24 h. TAN discs with persister-enriched MRSA biofilms were sonicated (**a**) in 0.9% saline after phage exposure to determine the amount of viable bacteria in CFU/mL or (**b**) in BHI prior to phage exposure. Results are from (**a**) three and (**b**) four independent experiments in triplicate. The mean CFU/mL and the 95% confidence intervals of the log-transformed data are indicated by the bar and the error bars, respectively.

Finally, as persisters are hard to eliminate with antibiotics^13,35^, the effect of ISP on persister-enriched MRSA biofilms was assessed using a validated model^31^. MRSA (LUH14616) within mature biofilms were exposed to high dose antibiotics (rifampicin/ciprofloxacin) for three consecutive days, reducing bacterial counts to a mean 3.7-log CFU/mL (95% CI 3.0–4.4) (Fig. 5a, Supplementary Table S5). Subsequent exposure of these persister-enriched mature biofilms to ISP (10^5^–10^8^ PFU/mL; MOI 100–10^5^) for 24 h did not affect bacterial counts (Fig. 5a, Supplementary Table S5). Instead, the persister-enriched population within mature biofilms increased with 3.5-log CFU/mL despite the presence of ISP. Also, mechanical disruption of persister-enriched mature biofilms did not improve the efficacy of ISP at 10^8^ PFU/mL (MOI 10^5^) (Fig. 5b, Supplementary Table S5). Reversible phage tolerance was observed within the phage-surviving persister population (data not shown). Thus, bacteria within and from persister-enriched mature biofilms tolerated ISP.

## Discussion

**Figure 6:**
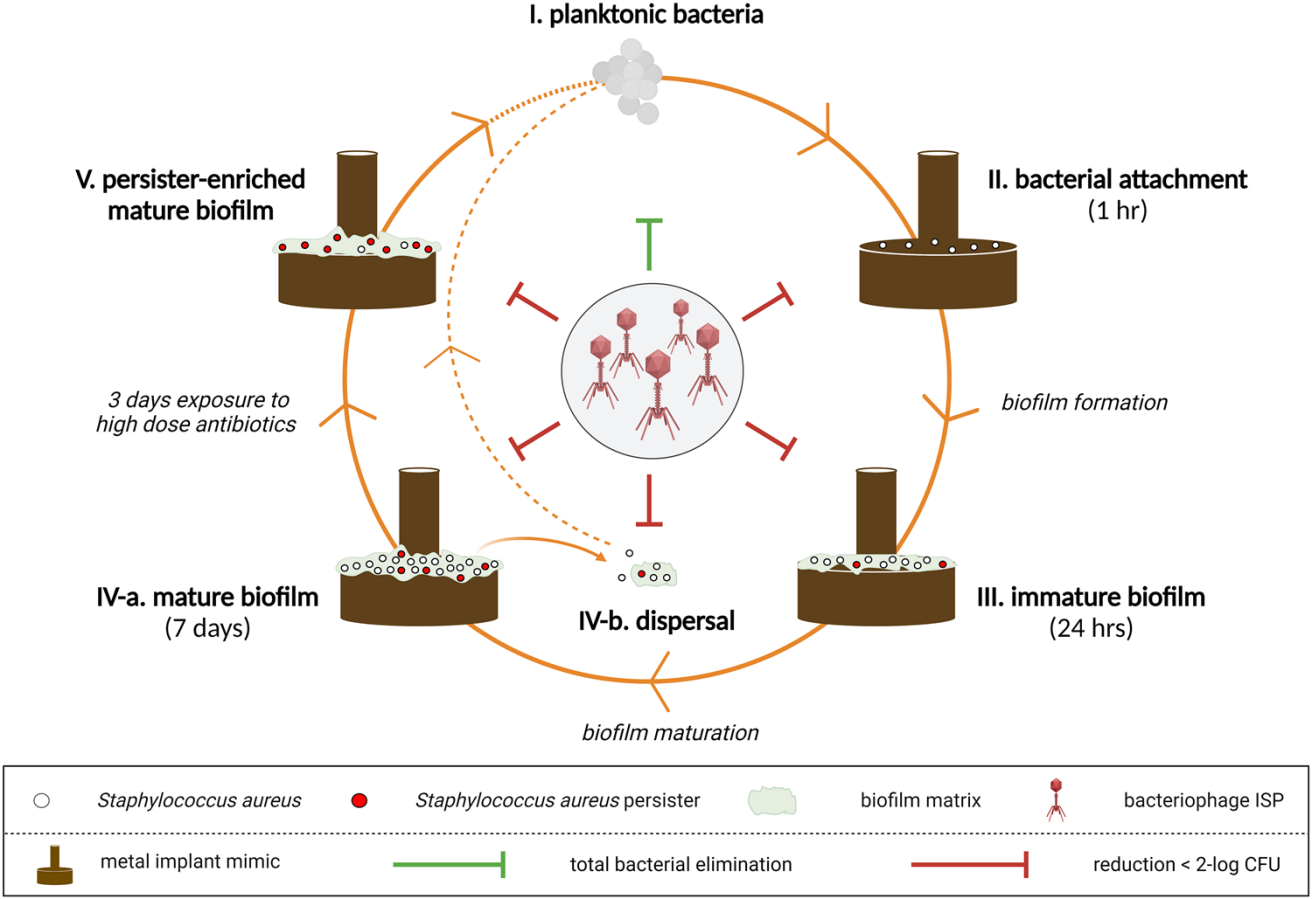
Overview of the effects of ISP on the different stages of the *S. aureus* life cycle. The effects of phage ISP were determined using different experimental in vitro models of *S. aureus*, including bacteria in (stage I) planktonic phase, (stage II) after 1 h attachment to the metal implant mimic, (stage III) within 24 h immature biofilms, (stage IV-a) within and (stage IV-b) dispersed from 7 day mature biofilms, and (stage V) within 7 day mature biofilms enriched for persisters by exposure to high dose antibiotics (rifampicin/ciprofloxacin) for three consecutive days. Titanium–aluminum–niobium (TAN) discs were used for bacterial attachment and biofilm culture. Created with BioRender.com.

The development of new therapeutic strategies to combat biofilm-associated infections is crucial due to lack of effective treatments and the worldwide threat of AMR^5,6^. In clinical practice, bacteriophages (phages) have been used to complement antibiotics as last resort treatment of biofilm-associated infections, as reported in case reports and small case series^23,36^. Comparing studies are lacking, and the absence of suitable controls hinders the interpretation of the efficacy of phages as single agents. Moreover, the effects of phages on the various stages of the bacterial life cycle are not well understood. To further elucidate this, we determined the effects of phage ISP against the different stages of the *S*. *aureus* life cycle in vitro (Fig. 6). The results clearly showed that ISP was highly effective on *S*. *aureus* in planktonic phase, while the efficacy of the phage decreased as the bacteria attached to the implant mimic and that there was no longer an effect of phages when bacteria were embedded in biofilms.

Regarding stage I, the results of our study show that ISP effectively eliminated all tested bacterial strains in planktonic phase. We used ammonium iron (II) sulphate hexahydrate (FAS) to neutralize residual phages to preclude overestimating the effect of phages in microbiological assays^29^. It should be noted that FAS did not affect CFU counting. ISP (*Kayvirus* genus of the Herelleviridae family, morphotype myovirus) binds to the bacterial wall-teichoic acid (WTA) of *S*. *aureus* and exclusively induces bacterial lysis. Moreover, the host range of ISP against *S*. *aureus* is broad^18^, which was confirmed by our study.

For stage II, bacterial attachment, we showed that the phage’s efficacy was considerably reduced against bacteria attached to the surface of the metal implant mimic. That is, ISP at high concentrations impaired the bacterial growth of attached bacteria compared to controls, but could not eliminate the bacterial fraction attached to the implant mimic. Whether this reduction of bacterial outgrowth is relevant in a clinical situation remains to be evaluated. Bacterial attachment affects the expression of many genes and is mediated by hydrophobic and electrostatic interactions that are predominantly induced by WTA in *S. aureus*^37^. While these factors improve the bacterial attachment to the implant surface, they might simultaneously affect phage adsorption by bacteria. Future studies involving techniques such as transcriptomics and proteomics are required to shed light on the mechanisms driving phage survival in the attached population. Chemical and biochemical techniques can be used to investigate whether bacterial cell-wall modifications occur upon bacterial attachment and how that contributes to impaired phage adsorption.

Stage III, the immature biofilm, is formed after bacterial attachment and the generation of microcolonies and EPS. We included a validated 7 day mature biofilm model (stage IV-a)^31^, which is considered more advanced, complex and clinically relevant than 24 h immature biofilms^25,26,38^. Thus far, we are not aware of other studies that have investigated the effects of phages on 7 day mature *S*. *aureus* biofilms. Due to the increased complexity of mature biofilms, we expected that *S*. *aureus* within mature biofilms would be less susceptible to ISP compared to immature biofilms. However, the effect of ISP on bacterial counts within immature as well as mature biofilms was limited, also after repeated exposure. Similar to our findings, phage ISP has been successful in the prevention of clinical *S*. *aureus* infection in an in vivo rabbit model, while being less successful as biofilm treatment^39^. Without proven exopolysaccharide depolymerase activity, it is unclear whether ISP is able to break-down the biofilm matrix^40^. We first hypothesized that the inefficacy of ISP could be due to the EPS hampering phage penetration into the biofilm and preventing phage adsorption by covering the bacterial cell wall. In agreement, we found the phage to be effective against bacteria after mechanical disruption of immature biofilms. Simultaneously, cross-sectional TEM analysis revealed the presence of phages and their interaction with bacteria throughout the intact biofilm, even in the deep biofilm layers. Our TEM analysis suggests a low phage-bacteria ratio, and thus a reduced chance of phage-infection, in the deep biofilm layers compared to the apical biofilm layer. Many bacteria were dividing throughout the biofilm, indicating that the metabolism of the biofilm-embedded bacteria may be sufficient to complete the lytic phage cycle. Alternatively, we reasoned that phage survival of the biofilm-embedded bacteria was due to the development of phage tolerance. Indeed, ISP was successful in eliminating the bacteria that survived ISP exposure within mature biofilms and that were allowed to recover in bacterial culture medium. This indicates a phage-tolerant, but not phage-resistant, phenotype. As the number of phage-surviving bacteria from mechanically disrupted mature biofilms of all strains was higher compared to that from immature biofilms, it could be that the phage-tolerant bacterial subpopulations expand upon biofilm maturation. ISP was more effective against mechanically disrupted (im)mature MSSA biofilms compared to mechanically disrupted (im)mature MRSA biofilms. This suggests that the limited phage efficacy against biofilm-embedded MRSA was mostly due to the presence of phage-tolerant bacteria rather than the biofilm-architecture. Mechanical disruption of the MSSA biofilms, on the other hand, considerably improved the efficacy of ISP, suggesting that the limited effect of ISP on intact MSSA biofilms was predominantly due to the biofilm-architecture.

Bacteria dispersed from mature MRSA, LUH15393 and LUH15395 biofilms (stage IV-b) were not affected by ISP, but incubation of these bacteria in bacterial culture medium before phage-exposure resulted in their switch to the planktonic, phage-sensitive phenotype. Of note, biofilm-dispersed MRSA required more time to switch to the phage-sensitive phenotype than the other strains. On the other hand, LUH15394 dispersed from mature biofilms were immediately eliminated by ISP, indicating that these bacteria reversed relatively faster to a phage-sensitive, planktonic phenotype after leaving the biofilm. Which factors triggered the time-dependent phenotypic switch from phage-tolerant to phage-sensitive, and reverse, is still unknown. Therefore, future experiments should focus on the defense mechanism of bacteria which makes them temporarily phage-tolerant, and whether this tolerance is phage-specific or more general in all biofilm-originating bacteria.

In stage V, persister-enriched mature biofilms were created to investigate the effects of ISP on bacterial subpopulations with lower metabolic activities, which are concomitant to biofilm maturation and exposure to antibiotics. In this context, previous recommendations have emphasized the importance of determining the efficacy of phages against bacteria with a reduced metabolic activity^41^. Previously, *S*. *aureus* persister cells exposed to Sb-1 were completely eliminated upon the reactivation of persisters^42^. We showed that stationary phase bacteria were eliminated by ISP, although relatively higher phage concentrations were required for elimination compared to mid-logarithmic phase bacteria. However, persisters within mature MRSA biofilms were not affected by ISP at such high concentrations. The persisters proliferated in the presence of ISP, highlighting factors other than metabolic activity being responsible for the phage tolerance. Nevertheless, it remains to be evaluated whether the mechanism driving phage tolerance in persisters is similar to that observed in the other stages of the *S. aureus* life cycle.

We acknowledge that this study has several limitations. First, despite the high-resolution and in-depth analysis of phage-exposed biofilms, our TEM analysis did not allow any quantification of the efficacy of ISP on bacteria within biofilms. In addition, as only a limited number of sections can be analyzed using TEM, visualization of the whole biofilm was not possible. The latter could be achieved by future studies using fluorescently labeled phages and visualizing the phage-bacteria interaction by confocal laser scanning microscopy. Second, generalizability of our results is not possible since we only investigated the effects of one phage (ISP). Third, clinical parameters like host-response, temperature, change of acidity of the clinical peri-implant tissue and environment were not taken into account for the phage-bacteria interaction. Nevertheless, ISP has shown to remain stable between pH 5–9 as well as temperatures ranging from 16, 37 and 42 °C^18^. It would be interesting to predict with our clinically relevant in vitro models whether ISP could improve the effects of antibiotics and the host-immune system to eradicate biofilm-associated infections in vivo.

The main conclusion of this study is that ISP was highly effective against *S*. *aureus* in planktonic phase, whereas the efficacy of the phage decreased as bacteria attached to the implant mimic and no relevant reduction by phages was observed on biofilm-embedded bacteria, including persisters. Importantly, we found that ISP could penetrate and interact with bacteria in biofilms, but that the biofilm architecture supported a phage-tolerant bacterial phenotype. Future experiments are required to investigate the mechanism underlying the observed phage-tolerance in our models. Phage therapy has a high potential for clinical application in a multimodality approach where also the biofilm integrity is addressed. Furthermore, adverse events during and after the use of phages is rare and phages can improve the efficacy of antibiotics^22–24^. Due to its broad-spectrum activity and our findings here, phage ISP as a single agent would only be effective in the most early stage of *S*. *aureus* infection and only against the planktonic bacterial subpopulation during any later stage of infection^39,43^.

### Supplementary Information

Supplementary Information.

## Data Availability

The data that support the findings of this study are available upon reasonable request from the corresponding author, M.V.
