# Supplementary material for: Bacteriophage ISP eliminates Staphylococcus aureus in planktonic phase, but not in the various stages of the biofilm cycle
Source: Sci Rep. 2024 Jun 22;14:14374. doi: 10.1038/s41598-024-65143-9 (PMC11193821; doi:10.1038/s41598-024-65143-9)
Supplement: Supplementary file 1 — Supplementary Information. [file 41598_2024_65143_MOESM1_ESM.pdf]

Supplementary figures and tables

Bacteriophage ISP eliminates *Staphylococcus aureus* in planktonic phase, but not in the various stages of the biofilm cycle

Mariëlle Verheul, Aat A. Mulder, Sven C.J. van Dun, Maia Merabishvili, Rob G.H.H. Nelissen, Mark G.J. de Boer, Bart G. Pijls, Peter H. Nibbering

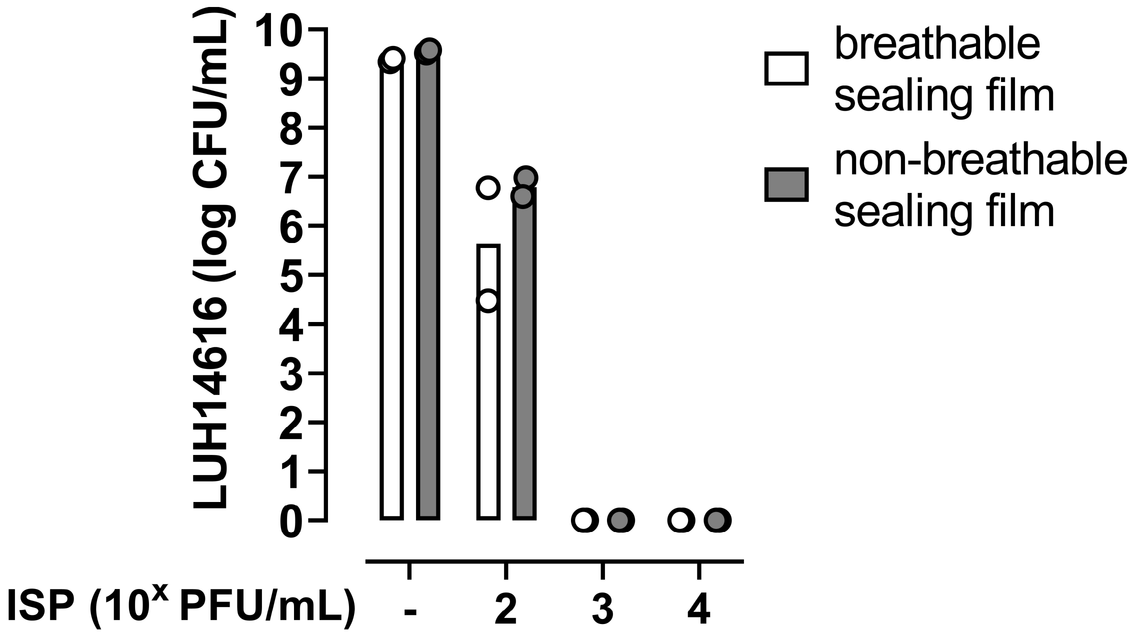

Supplementary Figure S1. Effect of the non-breathable plastic sealing film on the lytic activity of ISP. Methicillin-resistant *S. aureus* (MRSA; LUH14616) were brought to mid-logarithmic phase by incubation for 2.5 hrs in tryptic soy broth (TSB). MRSA were diluted to a final concentration of 10^6 CFU/mL and exposed to ISP at final concentrations ranging from $10^2 – 10^4$ plaque forming units (PFU)/mL (multiplicity of infection (MOI) $10^{-4} – 10^{-2}$) in brain-heart infusion (BHI) broth for 24 hrs in a 96-wells v-bottom polypropylene plate. The plate was sealed with either the non-breathable plastic sealing film or the breathable rayon sealing film. Residual phage activity was neutralized by the addition of 10 mM ammonium iron (II) sulfate hexahydrate (FAS) before microbiological determination of viable bacteria in CFU/mL. Results are from one experiment in duplicate. The mean CFU/mL is indicated by the bar.

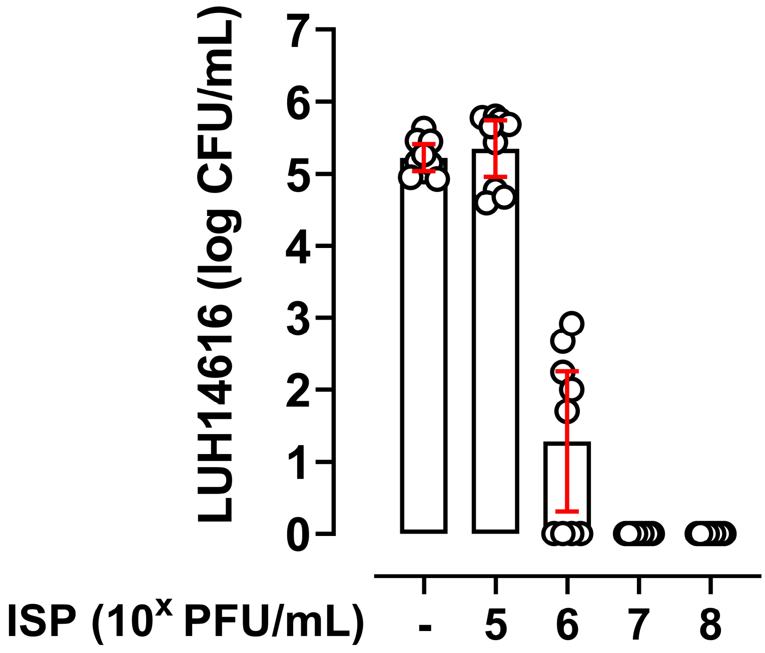

Supplementary Figure S2. Effect of ISP on planktonic methicillin-resistant *S. aureus* (MRSA) in stationary phase. MRSA (LUH14616) were brought to stationary phase by incubation for 24 hrs in tryptic soy broth (TSB), subsequently diluted to a final concentration of 10^6 CFU/mL and exposed to ISP at final concentrations ranging from 10^5 – 10^8 plaque forming units (PFU)/mL (multiplicity of infection (MOI) 0.1 – 100) in 0.9% saline spiked with 2% TSB for 24 hrs. Residual phage activity was neutralized by the addition of 10 mM ammonium iron (II) sulfate hexahydrate (FAS) before microbiological determination of viable bacteria in CFU/mL. Results are from 3 independent experiments each in triplicate. The mean CFU/mL and the 95% confidence intervals of the log-transformed data are indicated by the bar and the error bars, respectively.

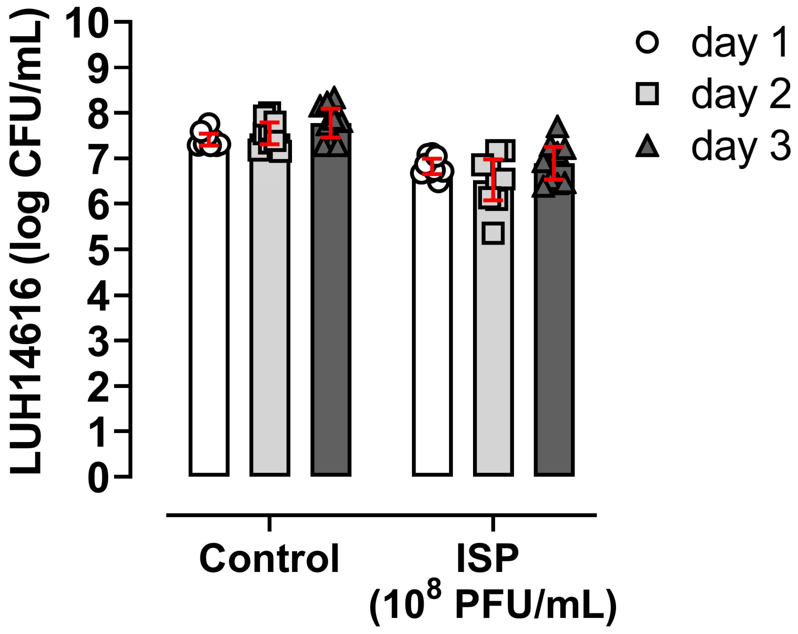

Supplementary Figure S3. Effect of ISP exposure for 3 consecutive days on methicillin-resistant *S. aureus* (MRSA) in mature biofilms. MRSA (LUH14616) in mature biofilms on titanium-aluminum-niobium (TAN) discs were exposed to ISP at 10^8 plaque forming units (PFU)/mL (multiplicity of infection (MOI) 1) in brain heart infusion (BHI) broth every 24 hrs for 3 consecutive days. Biofilms on TAN discs were sonicated in 0.9% saline and 10 mM ammonium iron (II) sulfate hexahydrate (FAS) to neutralize residual phage activity. Next, the bacterial load was determined microbiologically and depicted as CFU/mL. Results are from 3 independent experiments each in triplicate. The mean CFU/mL and the 95% confidence intervals of the log-transformed data are indicated by the bar and the error bars, respectively.

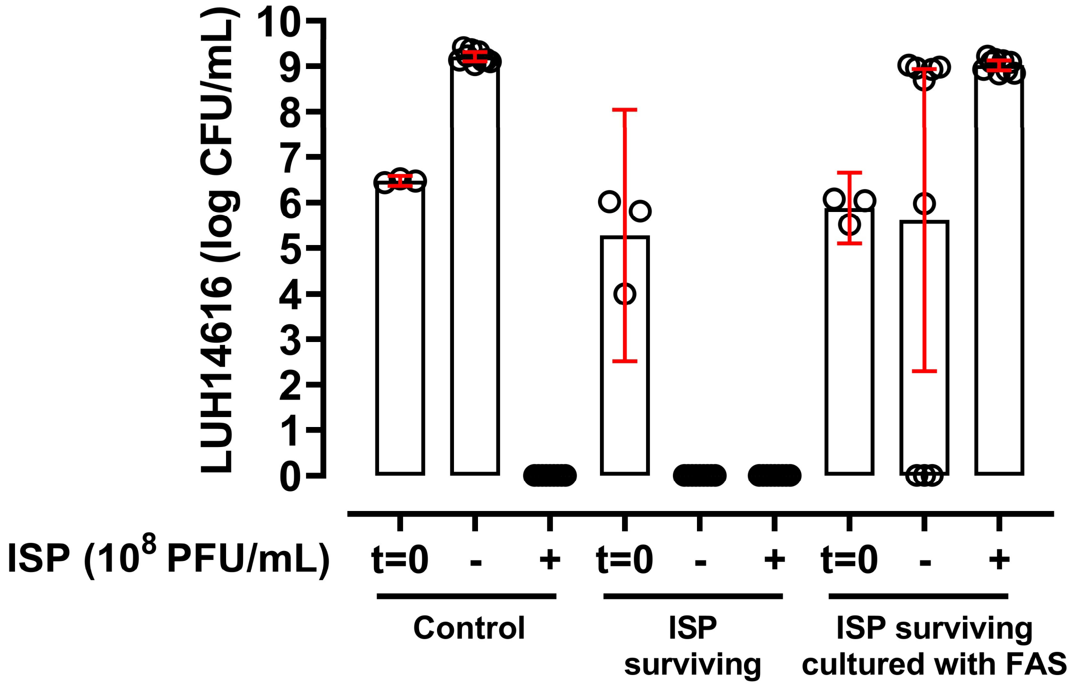

Supplementary Figure S4. Effect of ISP on re-cultured, mid-logarithmic methicillin-resistant *S. aureus* (MRSA) that survived exposure to ISP in mature biofilms. Colonies of MRSA (LUH14616) that survived ISP exposure (10^8 plaque forming units (PFU)/mL) within mature biofilms (ISP surviving) were re-cultured to mid-logarithmic phase. As control, such colonies were cultured in presence of 10 mM ammonium iron (II) sulfate hexahydrate (FAS) to neutralize residual phage activity (ISP surviving cultured with FAS). Colonies obtained from mature biofilms that were not exposed to ISP served as control (control). MRSA were diluted to a final concentration of 10^6 CFU/mL (t=0) and exposed to ISP at a final concentration of 10^8 PFU/mL (multiplicity of infection (MOI) 100) in brain heart infusion (BHI) broth for 24 hrs. Residual phage activity was neutralized by the addition of 10 mM FAS after 24 hrs phage exposure and before microbiological determination of viable bacteria in CFU/mL. Results are from 3 independent experiments each in triplicate. The mean CFU/mL and the 95% confidence intervals of the log-transformed data are indicated by the bar and the error bars, respectively.

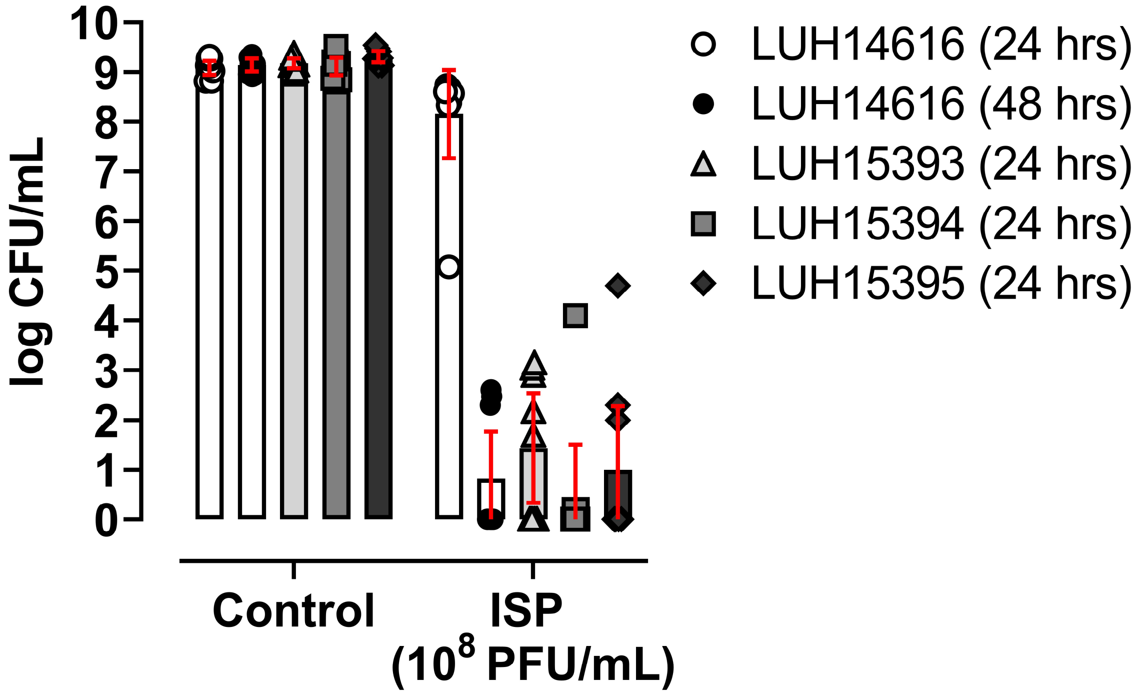

Supplementary Figure S5. Effect of pre-incubation of *S. aureus* dispersed from mature biofilms before exposure to ISP. Methicillin-resistant *S. aureus* (MRSA; LUH14616) and methicillin-sensitive *S. aureus* (MSSA; LUH15393, LUH15394 and LUH15395) dispersed from mature biofilms on titanium-aluminum-niobium (TAN) discs were incubated in brain-heart infusion (BHI) broth for 24 hrs (and 48 hrs for MRSA). Thereafter, bacteria were brought to a final concentration of 10^6 CFU/mL and exposed to phage ISP at a final concentration of 10^8 plaque forming units (PFU)/mL (multiplicity of infection (MOI) 100) in BHI for 24 hrs. Residual phage activity was neutralized by the addition of 10 mM ammonium iron (II) sulfate hexahydrate (FAS) after 24 hrs phage exposure and before microbiological determination of viable bacteria in CFU/mL. Results are from 3 independent experiments each in triplicate. The mean CFU/mL and the 95% confidence intervals of the log-transformed data are indicated by the bar and the error bars, respectively.

Supplementary Table S1. Mean log CFU/mL with 95% CI of the log-transformed data and mean difference (Δ mean) of *S. aureus* after 1 hr attachment (t=0) exposed to ISP

	bacteria after attachment (log CFU/mL)		
**strain**	**mean t=0**	**mean ISP (10^8 PFU/mL)**	**Δ mean**
LUH14616	**3.2**<br>(3.1-3.4)	**3.3**<br>(2.7-4.0)	**0.1**
LUH15393	**4.1**<br>(3.5-4.7)	**5.2**<br>(4.9-5.6)	**1.1**
LUH15394	**3.7**<br>(3.5-4.0)	**5.9**<br>(5.6-6.3)	**2.2**
LUH15395	**3.6**<br>(3.3-3.8)	**5.3**<br>(4.7-6.0)	**1.7**

Supplementary Table S2. Mean log CFU/mL with 95% CI of the log-transformed data and mean difference (Δ mean) of *S. aureus* in and from mechanically disrupted immature biofilms exposed to ISP.

	immature biofilm (log CFU/mL)			mechanially disrupted immature biofilm (log CFU/mL)		
**strain**	**mean control**	**mean ISP (10^8 PFU/mL)**	**Δ mean**	**mean t=0**	**mean ISP (10^8 PFU/mL)**	**Δ mean**
LUH14616	**7.3**<br>(7.0-7.5)	**6.4**<br>(6.0-6.8)	**-0.9**	**7.3**<br>(7.0-7.5)	**4.0**<br>(2.9-5.1)	**-3.3**
LUH15393	**7.4**<br>(7.2-7.5)	**7.5**<br>(7.3-7.6)	**0.1**	**7.4**<br>(7.2-7.5)	**1.2**<br>(0-2.5)	**-6.2**
LUH15394	**7.7**<br>(7.2-8.1)	**7.4**<br>(6.7-8.2)	**-0.2**	**7.7**<br>(7.2-8.1)	**0.4**<br>(0-1.3)	**-7.2**
LUH15395	**7.1**<br>(6.6-7.5)	**7.3**<br>(6.9-7.8)	**0.3**	**7.1**<br>(6.6-7.5)	**1.5**<br>(0.4-2.6)	**-5.6**

Supplementary Table S3. Mean log CFU/mL with 95% CI of the log-transformed data and mean difference (Δ mean) of *S. aureus* in and from mechanically disrupted mature biofilms exposed to ISP.

	mature biofilm (log CFU/mL)			mechanially disrupted mature biofilm (log CFU/mL)		
**strain**	**mean control**	**mean ISP (10^8 PFU/mL)**	**Δ mean**	**mean t=0**	**mean ISP (10^8 PFU/mL)**	**Δ mean**
LUH14616	**7.4**<br>(7.2-7.7)	**6.6**<br>(6.3-6.9)	**-0.8**	**7.4**<br>(7.2-7.7)	**8.5**<br>(8.2-8.9)	**1.1**
LUH15393	**8.2**<br>(7.9-8.4)	**7.8**<br>(7.6-7.9)	**-0.4**	**8.2**<br>(7.9-8.4)	**3.8**<br>(3.0-4.7)	**-4.3**
LUH15394	**8.3**<br>(8.0-8.5)	**7.7**<br>(7.2-8.1)	**-0.6**	**8.3**<br>(8.0-8.5)	**3.2**<br>(2.6-3.7)	**-5.1**
LUH15395	**8.3**<br>(7.9-8.6)	**8.1**<br>(8.0-8.2)	**-0.2**	**8.3**<br>(7.9-8.6)	**5.1**<br>(3.6-6.5)	**-3.2**

Supplementary Table S4. Mean log CFU/mL with 95% CI of the log-transformed data and mean difference (Δ mean) of *S. aureus* dispersed from mature biofilms exposed to ISP.

	bacteria dispersed from mature biofilm (log CFU/mL)								
	**mean t=0***			**mean ISP (10^8 PFU/mL)**			**Δ mean**		
	pre-incubation#			pre-incubation#			pre-incubation#		
**strain**	-	24 hrs	48 hrs	-	24 hrs	48 hrs	-	24 hrs	48 hrs
LUH14616	**6.6**<br>(5.6-7.5)	**6.2**<br>(5.7-6.7)	**6.0**<br>(5.6-6.3)	**8.7**<br>(8.5-8.9)	**8.2**<br>(7.3-9.0)	**0.8**<br>(0.0-1.8)	**2.1**	**1.9**	**-5.1**
LUH15393	**6.9**<br>(6.1-7.7)	**6.3**<br>(6.0-6.6)	-<br>-	**8.2**<br>(8.1-8.4)	**1.4**<br>(0.3-2.5)	-<br>-	**1.3**	**-4.9**	-
LUH15394	**7.0**<br>(5.7-8.3)	**6.2**<br>(5.7-6.7)	-<br>-	**1.7**<br>(0.0-3.8)	**0.5**<br>(0-1.5)	-<br>-	**-5.3**	**-5.7**	-
LUH15395	**6.4**<br>(4.9-7.9)	**6.2**<br>(5.9-6.5)	-<br>-	**8.5**<br>(8.4-8.6)	**1.0**<br>(0-2.3)	-<br>-	**2.1**	**-5.2**	-

* starting inoculum

[#] incubation of dispersed bacteria in brain-heart infusion (BHI) broth before exposure to control or ISP

Supplementary Table S5. Mean log CFU/mL with 95% CI of the log-transformed data and mean difference (Δ mean) of methicillin-resistant *S. aureus* in and from mechanically disrupted persister-enriched mature biofilms exposed to ISP.

	persister-enriched mature biofilm (log CFU/mL)			mechanially disrupted persister-enriched mature biofilm (log CFU/mL)		
**strain**	**mean control**	**mean ISP (10^8 PFU/mL)**	**Δ mean**	**mean control**	**mean ISP (10^8 PFU/mL)**	**Δ mean**
LUH14616	**3.7**<br>(3.0-4.4)	**7.2**<br>(6.4-7.9)	**3.5**	**3.4**<br>(3.1-3.7)	**5.6**<br>(3.6-7.7)	**2.2**
